# Evaluation of CareStart™ malaria HRP2/pLDH (Pf/PAN) combo rapid diagnostic test for diagnosis of *Plasmodium falciparum* infection in malaria co-endemic areas in association with parasite density

**DOI:** 10.1186/s12936-025-05276-y

**Published:** 2025-02-10

**Authors:** Michael Christian, Lenny Lia Ekawati, Aa Raka Pratama, Syavira Cahyaningati, Hermina K. Bere, Muhammad Rustam, Ichsan Kalbuadi, Jeltsin Andini, Jeng Yuliana, Ihsan Fadilah, Benedikt Ley, Kamala Thriemer, Ric N. Price, Inge Sutanto, J. Kevin Baird

**Affiliations:** 1 Oxford University Clinical Research Unit Indonesia, Faculty of Medicine Universitas Indonesia, Jakarta, Indonesia; 2 Department of Parasitology, Faculty of Medicine Universitas Indonesia, Jakarta, Indonesia; 3 Centre for Tropical Medicine & Global Health, Nuffield Department of Medicine, University of Oxford, Oxford, UK; 4 Menzies School of Health Research and Charles Darwin University, Darwin, Australia; 5 Mahidol-Oxford Tropical Medicine Research Unit, Faculty of Tropical Medicine Mahidol University, Bangkok, Thailand

**Keywords:** Malaria diagnosis, Rapid diagnostic test, RDT, *Plasmodium falciparum* malaria, Parasite density, Parasitaemia, HRP2, PLDH, Pf/Pan, CareStart^™^ Malaria HRP2/pLDH(Pf/PAN) Combo

## Abstract

**Background:**

As a widely accepted field standard diagnostic tool for malaria, microscopic examination is often difficult to perform in resource-poor settings. The immunochromatographic HRP2/pLDH (Pf/Pan) Rapid Diagnostic Tests (RDTs) serve as alternatives to microscopic examination for falciparum and non-falciparum malaria in co-endemic areas by detecting the histidine-rich protein 2 (HRP2) and pan-plasmodial lactate dehydrogenase (pLDH) antigen. However, Pf/Pan RDTs do not directly quantify parasitaemia. In this study, the diagnostic performance of Pf/Pan RDT and its association with parasite density was examined.

**Methods:**

Blood smears from patients who were screened for PRIMA Clinical Trial (Trial Registration Number: NCT03916003) conducted in East Sumba, Indonesia, and enrolled to its sub-study, ACROSS, were examined for microscopic examination and RDT using CareStart^™^ Malaria HRP2/pLDH (Pf/PAN) Combo (CareStart^™^ Pf/Pan RDT). Results were analysed for both diagnostic performance of RDT and its relationship with parasite density using a logistic regression model.

**Results:**

317 participants were included in this study and 158 (49.8%) were malaria positive by microscopy. Among all malaria-positive participants, *Plasmodium falciparum* infections accounted for 149 (94.3%) cases. The sensitivity and specificity of HRP2 band were 97.3% (95% CI 93.3–99.2) and 97.6% (95% CI 94.0–99.4), respectively, while that of pLDH band were 87.3% (95% CI 81.1–92.0) and 100% (95% CI 97.7–100). For each ten-fold increase in parasite density, the RDT had 12 times the odds of returning Pf/Pan-positive results (n = 126) compared to Pf-positive (n = 19) (OR: 12.1; 95% CI 5.18 to 34.8; p < 0.001).

**Conclusions:**

CareStart^™^ Pf/Pan RDT is reliable in diagnosing falciparum malaria and Pf/Pan-positive results indicate higher parasite density. Pf/Pan-positive results should alert the clinical staff of the increased risk of poor clinical outcome, and should be prioritized for microscopic examination compared to Pf-positive results.

**Supplementary Information:**

The online version contains supplementary material available at 10.1186/s12936-025-05276-y.

## Background

Despite vigorous global efforts, malaria morbidity and mortality burdens have not significantly diminished since 2015, with a 3% increase in incidence in 2020 and remained stable for the following three years [1]. Endemic nations face the impediment of limited resources in the remote rural settings where malaria burdens are heaviest. One of the greatest challenges in malaria management in such settings is reliable diagnosis. The field standard diagnostic method is blood film microscopic examination of peripheral blood [2, 3]. In expert hands, microscopy is highly reliable and provides identification of species as well as quantifying peripheral parasitaemia [2].

In constrained setting settings the use of microscopy for malaria diagnosis is difficult to implement and later to sustain at a good quality level. The accuracy of blood film microscopy relies heavily on the proficiency of the microscopist which requires intense training and certification. Reliable microscopic examination also requires a supportive working environment including access to clean slides, properly smeared, suitable staining chemicals, and a functioning quality microscope. Appropriate technical supervision, quality assurance, and refresher training are also required [2, 3]. In resource-poor settings without the ease of training and logistics, immunochromatographic malaria rapid diagnostic test (RDT) is enormously appealing [3].

There are many commercial brands and types of RDTs with a spectrum of costs, durability, and quality. The most widely used RDTs are HRP2-based RDTs, which detect histidine-rich protein 2 (HRP2) antigen that is specific to *Plasmodium falciparum*. However, this type of RDT has some limitations such as persistent positive result following a resolved infection caused by the presence of HRP2 antigen for an extended period, and its inability to detect other species of *Plasmodium* [4]. Another type of RDT is pLDH-based RDT, which detect pan-plasmodial lactate dehydrogenase (pLDH), antigen that occurs across all human plasmodial species (pan-plasmodial). Unlike HRP2, pLDH proteins stays present relatively shortly after a treated infection [4]. A study by Grandesso et al*.* in low-transmission setting showed that the median time for an individual to become pLDH-negative was two days after treatment intake, with 95% of cases became negative within 14 days. On the other hand, HRP2 stayed positive even after 42 days following treatment intake [5]. There are RDTs that utilize both antigens in one kit and allow the differential diagnosis of falciparum and non-falciparum malarias (Pf/Pan RDT), including CareStart^™^ Malaria HRP2/pLDH (Pf/PAN) Ag Combo RDT. It is affordable at USD 2–5 for a kit and its diagnostic performance has exceeded minimal sensitivity and specificity thresholds set by the World Health Organization (WHO) [6–10]. However, like other RDTs, Pf/Pan RDT cannot quantify the peripheral parasitaemia. Physicians and laboratorians involved in patient care rely on parasitaemia to assess the severity of infection, prognosis, and therapeutic response of the patient. Some studies associated test band intensity of the RDT readout to parasite density in blood, but this is limited to strong-line intensity only above the parasite density threshold of 1000 parasites/μL [11, 12].

Another approach to estimate parasitaemia using Pf/Pan RDT is by its positive-band pattern. The pLDH (Pan) band is less sensitive than that of HRP2 (Pf), requiring a higher parasite density to return positive result; a Pf-positive but Pan-negative test result could therefore suggest lower parasite densities compared to a result with two positive bands (Pf/Pan positive) [4, 7, 12]. There were a few studies that tried to see the association between RDT results and microscopic examination. A study by Martiáñez-Vendrell et al*.* in endemic and non-endemic countries demonstrated that pLDH concentrations had a greater correlation with *P. falciparum* with parasite density compared to HRP2 [13]. In Cameroon, Pf/Pan-positive patients had higher parasitaemia loads compared to who were only Pf-positive [14]. On contrary, a study by Diallo et al*.* in a low-transmission area in Senegal using CareStart^™^ Malaria HRP2/pLDH (Pf/PAN) Ag Combo RDT showed that the positivity rate of Pan band was low even at relatively high parasitaemia [8]. However, it was noted that the Pan band may also be more sensitive to degradation compared to the Pf band [8, 15, 16].

In the current study from a hypo-endemic setting in eastern Indonesia, the diagnostic implications of Pf-only positivity versus Pf/Pan positivity were examined in patients infected with *P. falciparum,* and their relationship with peripheral parasitaemia was assessed.

## Methods

### Study design and population

This cross-sectional study was a part of PRIMA (Trial Registration Number: NCT03916003)—a randomized controlled antimalarial clinical trial of patients with falciparum malaria—and a G6PD deficiency prevalence survey (ACROSS) that evaluated malaria point-of-care tests, conducted in East Sumba, Indonesia [17, 18]. In the current study, the samples used for screening blood smears and associated RDT (who enrolled to ACROSS following informed consents) results were analysed. Patients seeking care at the study clinics were eligible if they were resident for at least 12 months at of the study areas, were at least 12 months of age, had a fever (defined as axillary temperature ≥ 37.5 °C) or history of fever within the past 48 h, did not present clinical signs of severe malaria, and provided written informed consent. In the consent process, consent for minors was obtained from their parents or legal guardian, and minors above the age of 11 were also asked to provide written assent in addition to consent by their parents or legal guardian. Following screening, participants had a venous blood draw, from which blood smears and RDT tests were prepared. Participants were enrolled at four government clinics (Puskesmas) in the eastern and southern part of East Sumba, eastern Indonesia: Puskesmas Mangili, Baing, Tanaraing, and Melolo between April 2021 and March 2022. ACROSS aimed to enrol 160 malaria-positive participants (any species) and 160 malaria-negative participants.

### Microscopic examination as reference method

Thick and thin blood smears obtained from each participant were brought to the field laboratory located at Mangili and stained with Giemsa 3% for 40–50 min. Stained blood films were examined by WHO-standard certified expert (level 1) microscopist using standard oil-immersion 1000 × light microscopy (10 × ocular lens times 100 × objective lens). The threshold of detection for an expert microscopist examining a standard 100–200 fields of vision is 20 to 50 parasites/μL [3, 19, 20]. A smear was considered negative if no parasite was found after examining 200 high-power fields (HPFs) of thick blood film. Among positive smears, the microscopists recorded *Plasmodium* species, count, and stages of parasite identified in the examination. The number of parasites was counted against 200 white blood cells (WBCs) if 100 parasites or more had been found; or against 500 WBCs with fewer parasites. Parasites per microliter blood was calculated assuming a normal 8000 WBCs per μL of blood or 5000000 RBCs per μL of blood. The microscopists examining blood films did not know the parallel malaria rapid diagnostic test (RDT) result. All collected samples will undergo molecular analysis for species confirmation, HRP2 and HRP3 deletions, markers of drug resistance, and others and the results will be published in a separate article.

### HRP2/pLDH (Pf/Pan) antigen rapid diagnostic test

**Fig. 1:**
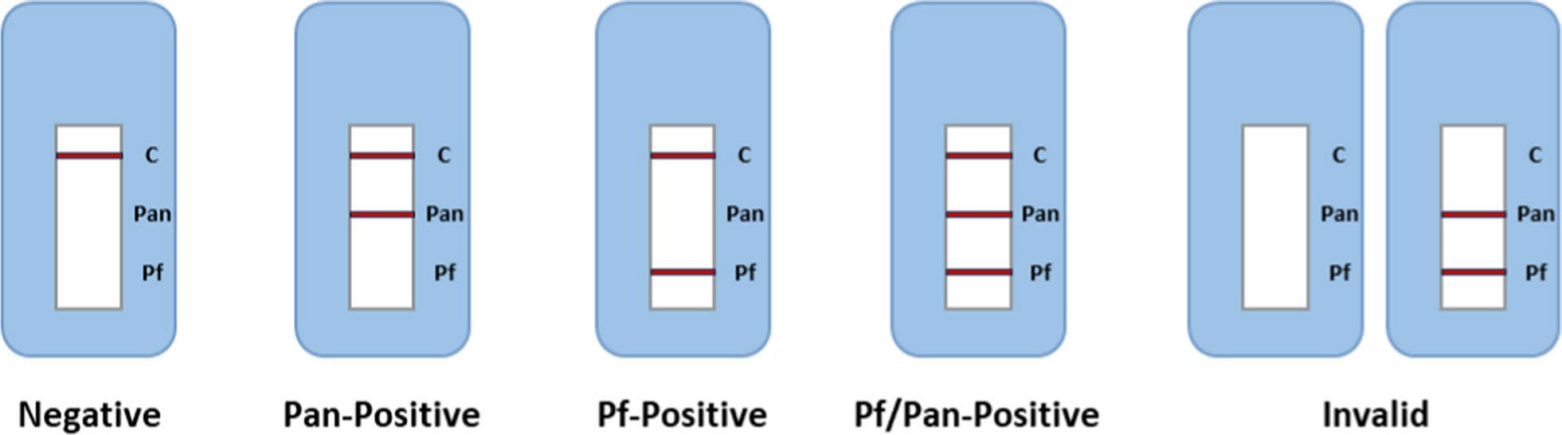
Schematic representation of interpretations of test outcomes with the Pf/Pan RDT kit. An HRP2-only positivity and pLDH-only positivity is referred to as Pf-positive and Pan-positive respectively, whereas the positivity of both bands is referred to as Pf/Pan-positive. Absence of C band indicates invalid result

Examination with RDT was performed immediately after the venous blood draw as a point-of-care test. The RDT used in this study was CareStart^™^ Malaria HRP-2/pLDH (Pf/Pan) Ag Combo Rapid Diagnostic Test (Accessbio/CareStart, ROK; CS-RDT), hereinafter referred to as CareStart^™^ Pf/Pan RDT. Three bands constitute the RDT kit: control (C), HRP2 (Pf), and pLDH (Pan). A valid negative result will have positive control band without positivity of either the HRP2 and or pLDH band, while a valid positive result will show a control band with either or both Pf and Pan bands. The test interpretation is schematically illustrated in Fig. 1.

The performance of CareStart^™^ Pf/Pan RDT is in line with WHO recommendations for the ability to detect falciparum and vivax infection in all transmission settings, the assay has a panel detection score of 75% at 200 parasites/μL, false positive results constitute less than 10% of all readings, and the invalid rate is less than 5% [2, 10]. Tests were performed in a well-lit room, and the results were read between 20 and 30 min after RDT loading according to manufacturer recommendation. The ambient temperature of RDT storage testing was less than 30 °C.

### Data analysis

RDT findings were interpreted by trained technicians (Fig. 1). Findings were evaluated against microscopically confirmed *P. falciparum* infection (both mono-infection and mixed-infection) as positive reference and participants without *P. falciparum* infection (both negative and non-falciparum infection) as negative reference. Sensitivity and specificity were calculated by comparing the counts of positive RDT results within true positive cases with all true positive cases and comparing the counts of negative RDT results within true negative cases with all true negative cases respectively (Table S2). Similarly, positive predictive value (PPV) and negative predictive value (NPV) were calculated by comparing the counts of true positive cases within RDT-positive results with all RDT-positive results, and the counts of true negative cases within RDT-negative results with all RDT-negative results respectively. McNemar’s Test was used to see if there is any significant difference in diagnostic performance between CareStart^™^ Pf/Pan RDT and microscopic examination by evaluating the discrepant test results between both modalities [21].

Participants with microscopy- and RDT-confirmed *P. falciparum* infection were further analysed to investigate the relationship of Pf-positive and Pf/Pan-positive RDT results (binary) with parasite density (continuous, asexual parasites per microlitre). Descriptive summaries of collected data were presented numerically and graphically to contrast the distribution of parasite density by test result. Binary logistic regression was used to model the probability of Pf/Pan positivity as a function of parasite density (log_10_ transformed). The linearity assumption was checked. Statistical analysis and data visualization were conducted using R Statistical Software (version 4.3.0). Code for logistic model fitting and visualization is available at https://github.com/ihsanfadil/prima-rdt.

### Ethical considerations

Ethical approval was obtained from the ethics committee of Faculty of Medicine Universitas Indonesia (approval number: 20-03-0370), Menzies School of Health Research (Approval number: 2019–2288), and Oxford Tropical Research Ethics Committee (Approval number: 65-19). Written informed consent was obtained from each study participant or their parent/legal guardian. Written informed assent was also obtained from children participants aged from 12 through 17 years old. Permission to carry out the study was also obtained from East Sumba Health Office and East Sumba Integrated Permit Office.

## Results

### Recruitment and baseline characteristics

**Fig. 2:**
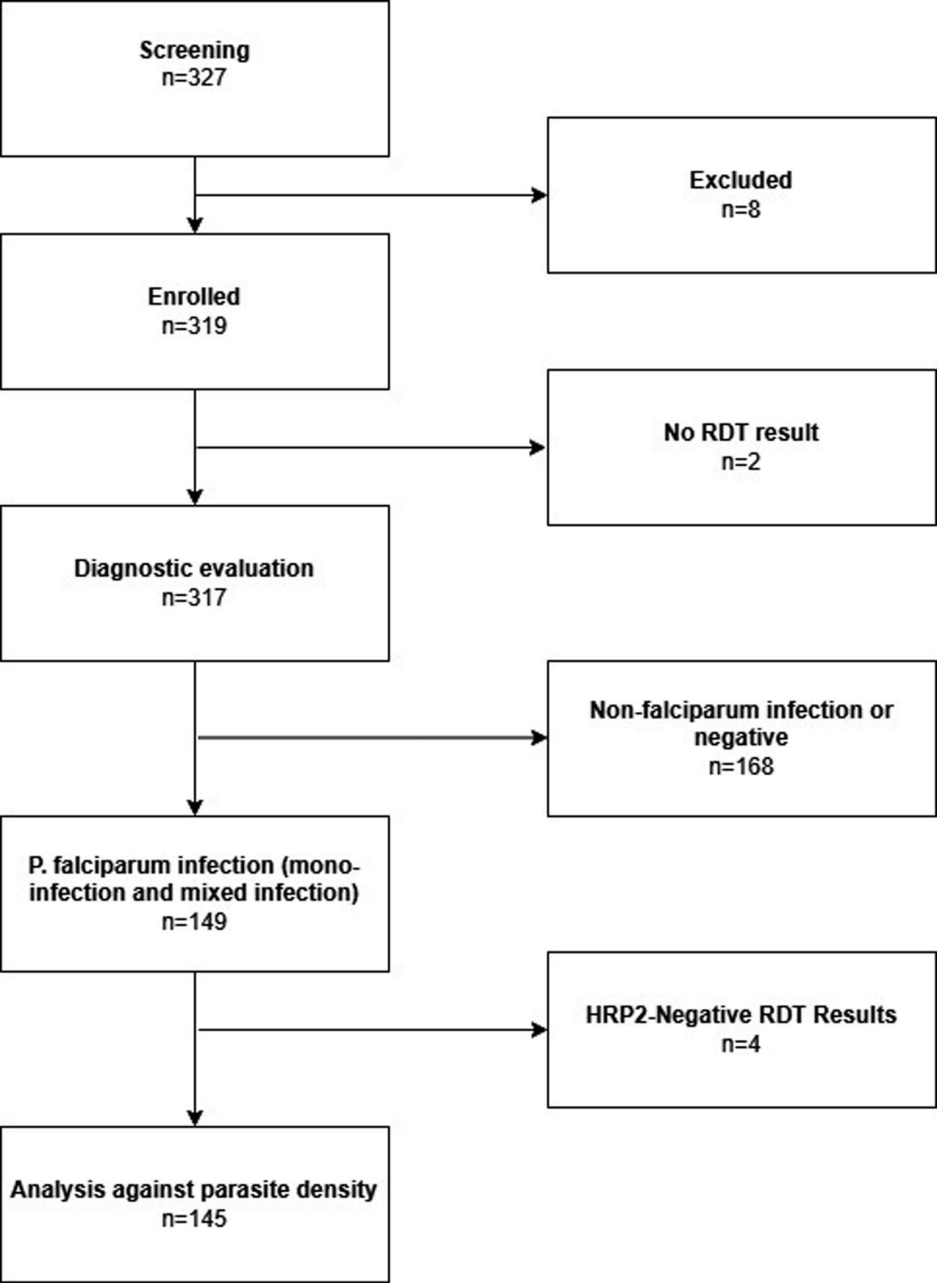
Participant selection flowchart*.* 327 individuals were screened and eight were excluded. Among the excluded, four were not enrolled due to operational and technical reasons, including limited personnel, which necessitated prioritizing the PRIMA clinical trial to ensure prompt treatments, and a shortage of point-of-care test kits at certain sites. Other reasons of exclusion include no consent, age less than 12 months, and the presence of clinical signs of severe malaria

In total 327 individuals were screened for enrolment and eight individuals were excluded. Two individuals declined informed consent, one presented with clinical sign of severe malaria, one was less than 12 months old, four others were not enrolled due to operational or technical reasons. Two participants were not RDT-tested and hence excluded from analysis. Among the enrolled 317 participants for RDT diagnostic evaluation, 149 (47%) were microscopically positive for *P. falciparum* infection, including two participants infected with mixed species. Four among those 149 participants returned either a valid negative (n = 1) or valid pan-positive RDT results (n = 3), and they were excluded from the analysis against parasite density (Fig. 2).

There were equal numbers of male and female participants. Most participants were between 10- and 26-years-old with more than 55% of total participants were under 18 years of age. Children under five years constituted 11% of all participants. In term of clinical presentation, 52% of participants presented with a documented fever at enrolment. The baseline characteristics of the participants are summarized in Table 1.

**Table 1:** Baseline characteristics of the participants

Characteristic	Values
Number of participants	
Counts (%)	317 (100)
Age in years	
Median (Q1-Q3)	16 (9–28.5)
Range (Minimum–maximum)	79 (1–80)
Age group	
< 5 years old counts (%)	36 (11.4)
5–17 years old counts (%)	140 (44.2)
≥ 18 years old counts (%)	141 (45.4)
Sex	
Females counts (%)	161 (50.8)
Males counts (%)	156 (49.2)
Measured fever	
Participants with measured fever counts (%)	166 (52.4)

*Q1* first quartile, *Q3* third quartile

### Microscopic examination results

Overall, 158 participants tested positive for malaria. *P. falciparum* infections occurred in 149 (94.3%) of positive participants, including two mixed-species infection cases: One *P. falciparum* and *Plasmodium vivax* mixed infection, and one *P. falciparum* and *Plasmodium malariae* mixed infection. The remaining malaria-positive participants were seven with *P. vivax* and two with *P. malariae* infections.

The geometric mean parasitaemia was 7575 parasites/µL (Geometric standard deviation (GSD) 5.76) and the median was 11000 parasites/µL (Q1-Q3 3721–22500) respectively. Five individuals had parasite densities greater than 100,000 parasites/µL, four of whom were children. These participants were included in the analysis as there was no clinical sign of severe disease. Both the proportion of cases and parasitaemia were higher in children (defined as less than 18 years of age) compared to adults (Table 2). The microscopic results of all examined blood smears are available in Table S1.

**Table 2:** Characteristics of *P. falciparum* infections by age group

Characteristic	< 5 y.o	5 to 17 y.o	≥ 18 y.o	Total	p value
Total	7	84	58	149	
Infection types (case counts)					
Mono-infections (%)	7 (4.7)	83 (55.7)	57 (38.2)	147 (98.6)	0.684*
Mixed Pf-Pv infections (%)	0 (0.0)	1 (0.7)	0 (0.0)	1 (0.7)	
Mixed Pf-Pm infections (%)	0 (0.0)	0 (0.0)	1 (0.7)	1 (0.7)	
Gametocytes (case counts)					
Present (%)	1 (0.7)	17 (11.4)	5 (3.4)	23 (15.4)	0.11*
Not present (%)	6 (4.0)	61 (40.9)	51 (34.2)	118 (79.2)	
Missing (%)	0 (0.0)	6 (4.0)	2 (1.3)	8 (5.4)	
Parasite Density (parasites/µL)					
Geometric mean (GSD)	10866 (6.97)	8698 (5.65)	5937 (5.81)	7575 (5.76)	0.381**
Median (Q1-Q3)	19000 (4628–40500)	12027 (4516–26500)	8198 (2572–16000)	11000 (3721–22500)	
Minimum–maximum	298–90000	127–259216	46–921500	46–921500	
Parasite density > 100,000 (%)	0 (0.0)	4 (2.7)	1 (0.7)	5 (3.4)	

Proportion of infection types, types of readings, gametocytes, and parasite density > 100,000 are expressed in case counts (%). Geometric mean, median, and minimum and maximum parasite densities are expressed in parasites/µL. Proportion of cases were calculated against total *P. falciparum* cases. *Q1* first quartile, *Q3* third quartile, *GSD* geometric standard deviation, *Performed with fisher exact, **Performed with One-way ANOVA on log-transformed values

### Pf/Pan rapid diagnostic test performance

The RDT findings concurred with microscopy (Table 3). Among the 149 microscopy-positive *P. falciparum* cases, all but four were registered as Pf/pan-positive or Pf-positive by RDT. Three of those four were pan-positive and one was negative. Among the nine non-falciparum malaria microscopy-positives (seven *P. vivax* and two *P. malariae* cases), all registered as pan-positive. Lastly, among microscopy negatives, four were positive by RDT, and all among those four were Pf-positive.

**Table 3:** RDT results (%) based on the type of malaria infections

		Microscopic examination					
		Pf (n = 147)	Pf-Pv (n = 1)	Pf-Pm (n = 1)	Pv (n = 7)	Pm (n = 2)	Neg (n = 159)
RDT	Pf/Pan	124 (39.1)	1 (0.3)	1 (0.3)	0 (0.0)	0 (0.0)	0 (0.0)
	Pf	19 (6.0)	0 (0.0)	0 (0.0)	0 (0.0)	0 (0.0)	4 (1.3)
	Pan	3 (0.9)	0 (0.0)	0 (0.0)	7 (2.2)	2 (0.6)	0 (0.0)
	Negative	1 (0.3)	0 (0.0)	0 (0.0)	0 (0.0)	0 (0.0)	155 (48.9)

For microscopic examination, n: total number diagnosed by microscopy for each category, *Pf P. falciparum* mono-infection, *Pf-Pv P. falciparum* and *P. vivax* mixed infection, *Pf-Pm P. falciparum* and *P. malariae* mixed infection, *Pv P. vivax* mono-infection, *Pm P. malariae* mono-infection, *Neg* negative microscopic results. Percentages are calculated against the total number diagnosed by microscopic examination across all categories

The sensitivity and specificity of the HRP2 band (Pf- and Pf/Pan-positive) in *P. falciparum* infection diagnosis were 97.3% (95% confidence interval (95%CI) 93.3–99.3) and 97.6% (95%CI 94.0–99.4), respectively. The positive predictive value (PPV) and negative predictive value (NPV) were 97.3% (95%CI 93.2–99.0) and 97.6% (95%CI 94.0–99.0) respectively. No significant difference was found between microscopic examination and HRP2 band results in detecting falciparum cases (McNemar’s Test, p = 1.000).

On the other hand, the sensitivity and specificity of the pLDH band in malaria diagnosis were 87.3% (95%CI 81.1–92.0) and 100% (95%CI 97.7–100), respectively. The PPV and NPV were 100% (95%CI 97.4–100) and 89.1% (95%CI 84.5–92.5), respectively. There was a difference in the diagnostic performance between microscopic examination and pLDH band, with microscopic examination detected more positive malaria cases (McNemar’s Test, p < 0.01) compared to CareStart^™^ Pf/Pan RDT.

For notes, the PPV and NPV reported here has a limited interpretation since the true prevalence is not known in this study, given the participants selection process of the ACROSS study that specifically aimed to enrol 160 malaria-positive participants and 160 malaria-negative participants.

### Association between falciparum-indicative RDT results and parasite density

**Fig. 3:**
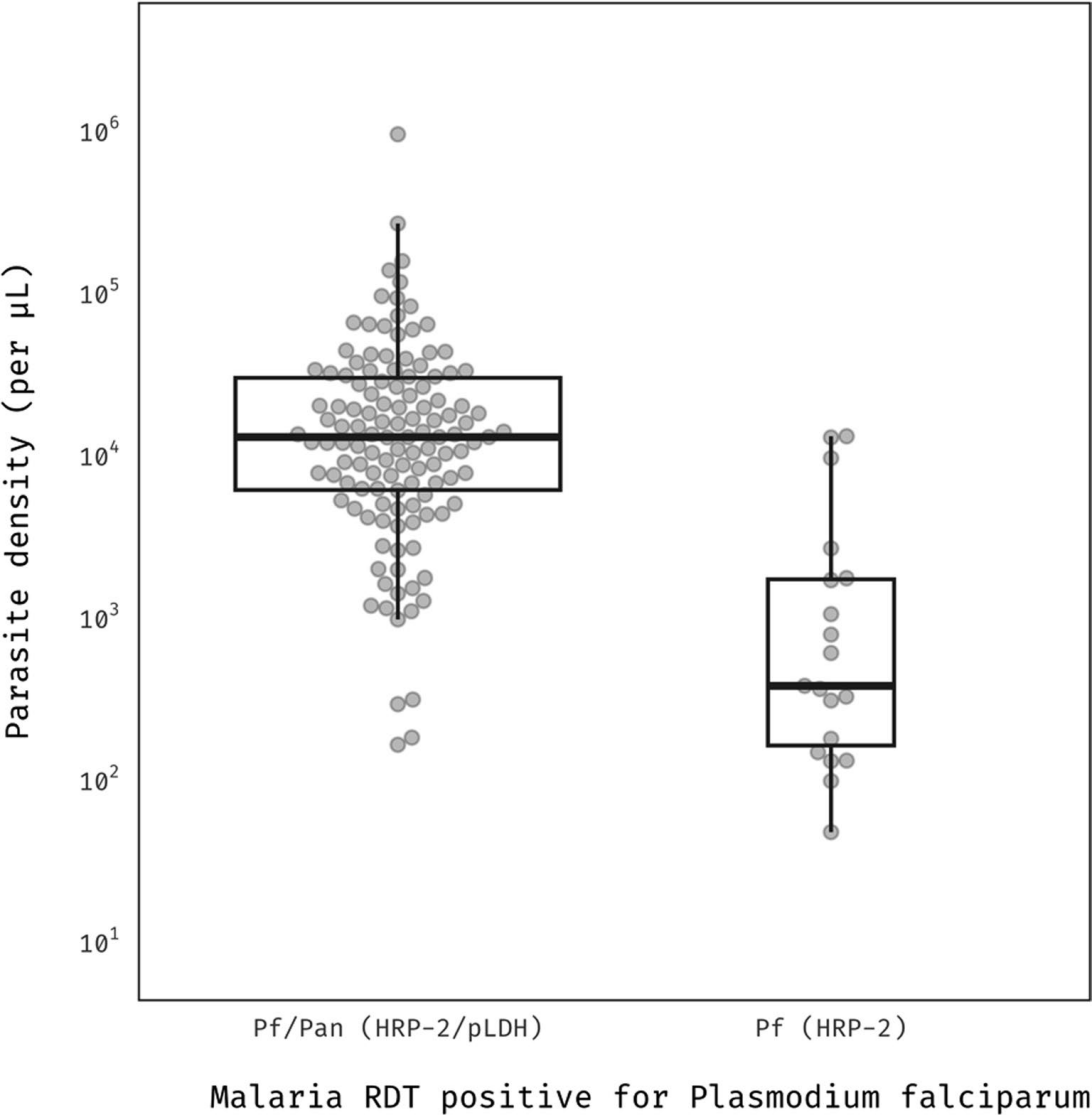
*Plasmodium falciparum* density by test result. Box width is proportional to the square root of the number of participants. Vertical axis is shown on the base10 logarithmic scale

A subgroup analysis was conducted on 126 participants with Pf/Pan-positive results and 19 participants with Pf-positive results to explore the association between parasitaemia and RDT positivity in participants with falciparum-indicative RDT results. Pf/Pan-positive participants exhibited higher parasite densities compared to those who were Pf-positive, as depicted in Fig. 3 and Table S3.

**Fig. 4:**
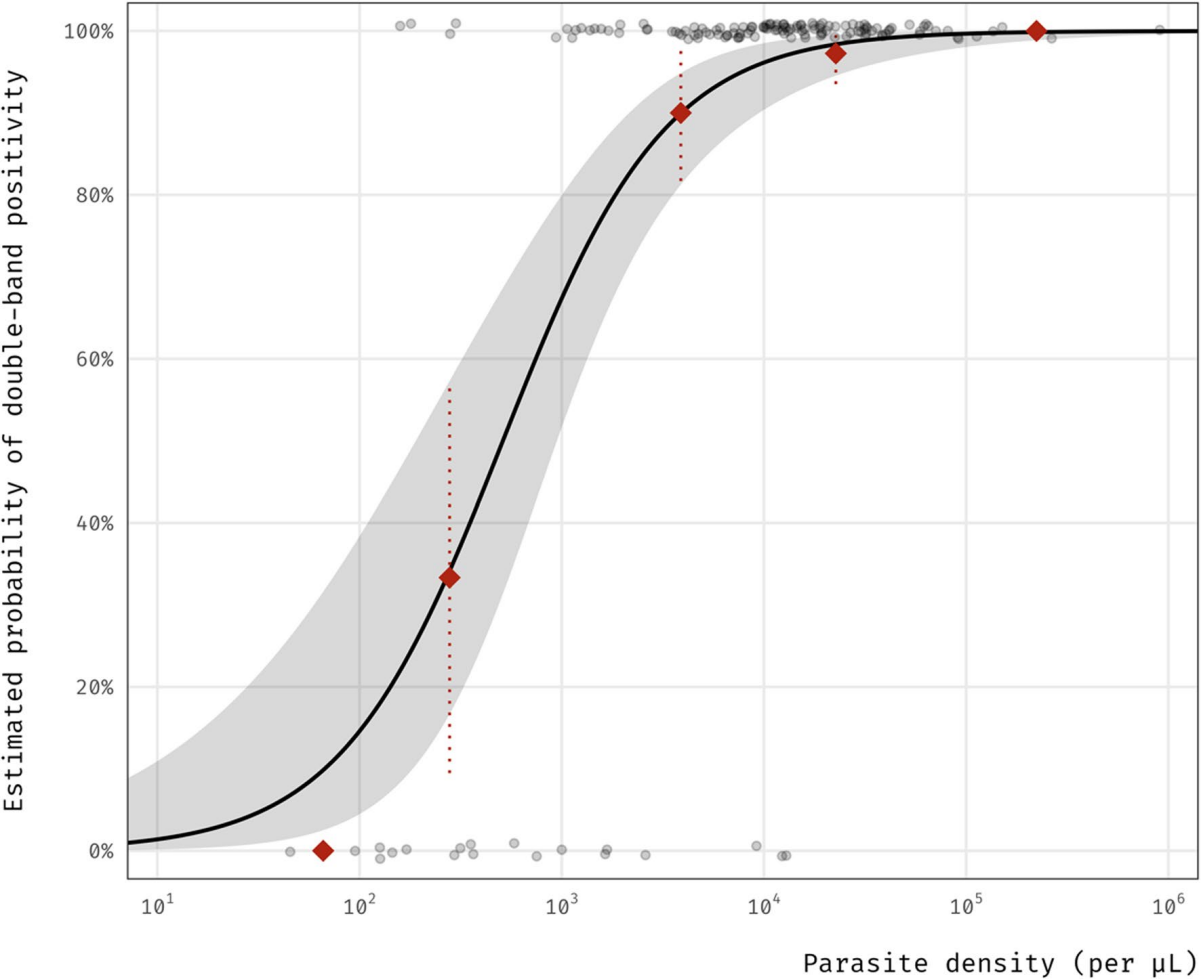
Estimated probabilities of testing HRP2/pLDH (+ / +) in participants with *P. falciparum* mono- or mixed-infection across parasite density levels. Grey bubble denotes the observed test result data (jittered). Red diamond indicates the proportion of participants testing Pf/pan positive by parasite density group (lowest = [10^1^, 10^2^] to highest = [10^5^, 10^6^]), where the location of the diamond along the horizontal axis represents the geometric mean of parasite density within each group). Dotted line indicates the 95% confidence interval around the group proportion. Grey area indicates the 95% confidence interval around the point probability estimate (black curve). Horizontal axis is shown on the logarithmic scale

There was a positive association between parasite density and Pf/pan positivity. A tenfold increase in *P. falciparum* density was associated with an estimated 12-fold times in the odds of RDT to return Pf/pan-positive results compared to Pf-positive (Odds ratio (OR) = 12.1; 95% CI 5.18 to 34.8; p < 0.001). On the probability scale, the same increase was associated with an up to 62% increase in the probability of Pf/pan positivity. Figure 4 illustrates the estimated probabilities for testing Pf/pan positive as parasite density increases. There was no clear evidence of non-linearity (Fig. S2).

## Discussion

CareStart^™^ Pf/Pan RDT band-positivity patterns were associated with the parasite density of *P. falciparum* mono-infections. Pf/Pan-positive results occurred with all parasitaemia ≥ 13000/µL, whereas most (13 of 19) Pf-positive results occurred when parasitaemia was < 1000/µL. The two mixed infections observed by microscopy were Pf/Pan-positive, accounting for only 2% of Pf/Pan-positive results. In the study population, Pf/Pan-positive RDT findings are associated with relatively higher *P. falciparum* parasitaemia compared to Pf-positive RDT. The only exceptions to this rule were four RDT Pf-positives that were microscopy-negative, along with the minority of cases (6 of 19) having > 1000/µL. The presence of both pLDH and HRP2 band indicates higher parasite density in falciparum infections compared to HRP2 only.

The findings in this study are consistent with those from previous studies, where the pLDH band required higher parasite densities to return a positive result, regardless of the species [4, 7, 12, 22, 23]. Patients presenting Pf/pan-positive RDT should be monitored more vigilantly due to being at risk of high-grade parasitaemia, which is associated with poorer outcomes and increased risk of mortality [2, 24–26]. Conversely, Pf-positive RDT results appear consistent with low-grade and relatively less threatening parasitaemia.

Pf/pan-positivity may also occur in mixed infections and that diagnosis carries important implications regarding chemotherapeutics strategies. The proportion of these representing mixed infections rather than higher-grade *P. falciparum* parasitaemia was very low in the setting of this study, but this may vary widely among sites. In additional vigilance, managing these patients may include a microscopic examination for possible mixed species infection to decide on chemotherapeutic regimen.

There were *P. falciparum* microscopy-positive infections that did not return Pf-positive RDT results. These cases consisted of three Pan-positive results and one negative result. The range of parasite densities of these cases ranged between 127 and 12,500 parasites/µL. The absence of pLDH band in the negative result can be easily explained by the relatively low corresponding parasite density at 127 parasites/µL. However, for the absence of HRP2 band, there were three possible explanations which are faulty kits, human error, or HRP2 gene deletion [27]. Although care was taken during the storage and transport of RDT kits, there was still a possible damage in few instances, especially when the temperature was higher than average and RDT was exposed to heat for a longer period of time. This exposure could lead to poor performance of RDT in detecting parasite antigen (false negative result) even if the control band was visible [28, 29]. On the other hand, prozone effect—which is associated with high concentration of antigen—is not considered a possible explanation, given the low parasite density of the cases [30, 31]. Finally, HRP2 gene deletion as a possible cause of a very small proportion of false negative HRP2 band findings cannot be ruled out although there has not been any report from the area to date. This finding, nevertheless, should raise a suspicion of such occurrences in the area, and even prompt further evaluation by future investigators, as it will potentially affect the performance of RDT in the corresponding area.

## Conclusion and recommendation

The findings in this study suggest that CareStart™ Pf/Pan RDT is a reliable diagnostic tool in diagnosing falciparum malaria, with the pLDH band positivity together in the presence of HRP2 band (Pf/Pan positivity) in any test result is indicative of higher parasite density. This finding implies that malaria cases with Pf/pan-positive result should alert the clinical staff of the risk that the patient may have a high parasitaemia and thus an increased risk of poor outcome. Furthermore, the inability of the RDT to distinguish *falciparum* mono-infection from mixed infection means that a further diagnostic evaluation is needed to respond to Pf/Pan-positive RDT result. An RDT-diagnosed malaria case that returns Pf/Pan-positive result should be prioritized for microscopic examination, as it can confirm both the parasite density and the plasmodium species in the infection. Alternatively, other types of RDTs that could differentiate between species such as CareStart™ Malaria Pf/Pv Combo test [33] or CareStart™ Malaria Pf/VOM combo [34].

## Limitation

The study design of ACROSS sought to enrol 160 malaria-positive participants and 160 malaria-negative participants. Among the malaria-positive participants, 98 participants from PRIMA with *P. falciparum* mono-infections were included. There were positive malaria cases of any species found during screening that were not enrolled following the achievement of enrolment target in the malaria-positive group, and only few non-falciparum and mixed infection cases were captured although the ratio between *P. falciparum* and *P. vivax* cases was estimated at 75:15. Consequently, the prevalence of malaria and true distribution of malaria species in the study area could not be reflected in this study.

Secondly, quantitative real-time polymerase chain reaction (qPCR) was not performed to confirm the diagnosis of malaria. It leads to potential overestimation or underestimation of true malaria occurrence since the diagnosis was only made based on light microscopy. Although the central laboratory is capable to perform qPCR, this study was not funded to carry one out. However, all collected samples will undergo molecular analysis for species confirmation, HRP2 and HRP3 deletions, markers of drug resistance, and others and the results will be published in a separate article.

Lastly, the study was conducted in a hypo-endemic area with a specific malaria transmission dynamic. Therefore, some of the findings in this study might not be applicable to other geographical settings and transmission levels. Further study with a larger scope might be needed.

## Supplementary Information

Additional file 1: Table S1. Results of microscopic examination on all individuals enrolled in ACROSS Study. Table S2. CareStart^TM^ Pf/Pan RDT Test results against microscopic examination. Table S3. Characteristics of individuals with *P. falciparum *infections based on RDT results. Figure S1. Distributions of parasite density by test result.the original scale andlogarithmic scale. The inset plot in, a participant with a high parasite density of almost 1000000 per microlitre is shown. Figure S2. Approximately linear relationship between log_10_-parasite density and the log-odds of Pf/pan positivity. The model was fitted using a logistic regression model that allows for non-linear relationship using restricted cubic splines with three knots. Grey area indicates the 95% confidence interval around the estimated log-odds. Horizontal axis is shown on the logarithmic scale.

## Data Availability

The dataset used for analysis in the current study are available from the corresponding author on reasonable request.

## Abbreviations

CI: Confidence interval
CS Pf/Pan RDT: CareStart^™^ Malaria HRP-2/pLDH (Pf/PAN) Combo RDT
HRP2: Histidine-Rich Protein 2
OR: Odds ratio
Pf: Plasmodium falciparum
pLDH: Pan-plasmodium Lactate Dehydrogenase
Pm: Plasmodium malariae
Pv: Plasmodium vivax
RDT: Rapid Diagnostic Test
