# Supplementary material for: Evaluation of CareStart™ malaria HRP2/pLDH (Pf/PAN) combo rapid diagnostic test for diagnosis of Plasmodium falciparum infection in malaria co-endemic areas in association with parasite density
Source: Malar J. 2025 Feb 10;24:39. doi: 10.1186/s12936-025-05276-y (PMC11812152; doi:10.1186/s12936-025-05276-y)
Supplement: Supplementary file 1 — Additional file 1: Table S1. Results of microscopic examination on all individuals enrolled in ACROSS Study. Table S2. CareStartTM Pf/Pan RDT Test results against microscopic examination. Table S3. Characteristics of individuals with P. falciparum infections based on RDT results. Figure S1. Distributions of parasite density by test result.the original scale andlogarithmic scale. The inset plot in, a participant with a high parasite density of almost 1000000 per microlitre is shown. Figure S2. Approximately linear relationship between log10-parasite density and the log-odds of Pf/pan positivity. The model was fitted using a logistic regression model that allows for non-linear relationship using restricted cubic splines with three knots. Grey area indicates the 95% confidence interval around the estimated log-odds. Horizontal axis is shown on the logarithmic scale. [file 12936_2025_5276_MOESM1_ESM.docx]

**Table S1. Results of microscopic examination on all individuals enrolled in ACROSS Study.**

|  |  | **Parasite Density** |  |  |
| --- | --- | --- | --- | --- |
| **Microscopic Results** | **Positive (%)** | **Geometric mean (GSD)** | **Median (Q1-Q3)** | **Min-max** |
| Any species | 159 (49.8) | 7303 (5.7) | 10500 (2661-20000) | 46-921500 |
| *P. falciparum* | 148 (46.4) | 7454 (5.8) | 10635 (3625-21750) | 46-921500 |
| *P. vivax* | 7 (2.2) | 3864 (4.1) | 7794 (1650-11181) | 457-15414 |
| *P. malariae* | 2 (0.6) | 4425 (3.4) | 6183 (1865-10500) | 1865-10500 |
| Pf-Pv mixed* | 1 (0.3) | 40451 (-) | - | - |
| Pf-Pm mixed* | 1 (0.3) | 15267 (-) | - | - |

The proportion of readings done on thin films were counted based on positive results of any and each species. The parasite density of pf-pv and pf-pm mixed infections were 40451 and 15267 respectively.

**Table S2. *CareStart*^TM^ Pf/Pan RDT Test results against microscopic examination.**

| a) |  | **Microscopic Pf positive** | **Microscopic Pf negative** | **Total** |
| --- | --- | --- | --- | --- |
|  | **HRP2 positive** | 145 | 4 | 149 |
|  | **HRP2 negative** | 4 | 164 | 168 |
|  | **Total** | 149 | 168 | 317 |

| b) |  | **Microscopic malaria positive** | **Microscopic malaria negative** | **Total** |
| --- | --- | --- | --- | --- |
|  | **pLDH positive** | 138 | 0 | 138 |
|  | **pLDH negative** | 20 | 159 | 179 |
|  | **Total** | 158 | 159 | 317 |

a) HRP2-band positivity against falciparum cases detected by microscopic examination; and b) pLDH-band positivity against all malaria cases detected by microscopic examination.

**Table S3. Characteristics of individuals with *P. falciparum* infections based on RDT results**

| **Variables** | **Pf-positive** | **Pan-positive** | **Pf/Pan-positive** |
| --- | --- | --- | --- |
| **Age in years** |  |  |  |
| Median (Q1-Q3) | 18 (9.5-33) | 20 (18-25) | 15 (10-26) |
| **Sex** |  |  |  |
| Male sex counts (%) | 10 (52.6) | 2 (66.7) | 70 (55.6) |
| **Measured fever** |  |  |  |
| Participants with measured fever counts (%) | 9 (47.4) | 3 (100) | 70 (55.6) |
| **Infection Type** |  |  |  |
| Pf mono-infection counts (%) | 19 (100) | 3 (100) | 124 (98.4) |
| Mixed infection counts (%) | 0 (0) | 0 (0) | 2 (1.6) |
| **Types of reading** |  |  |  |
| Thick film counts (%) | 19 (100) | 1 (33.3) | 54 (42.9) |
| Thin film counts (%) | 0 (0) | 2 (66.7) | 72 (57.1) |
| **Parasite Density** |  |  |  |
| Geometric mean (GSD) | 615 (5.3) | 6719 (2.7) | 11459 (4.0) |
| Median (Q1-Q3) | 367 (158-1667) | 11500 (6805-12000) | 12500 (5859-29500) |
| Maximum-minimum | 46-12647 | 2110-12500 | 159-921500 |


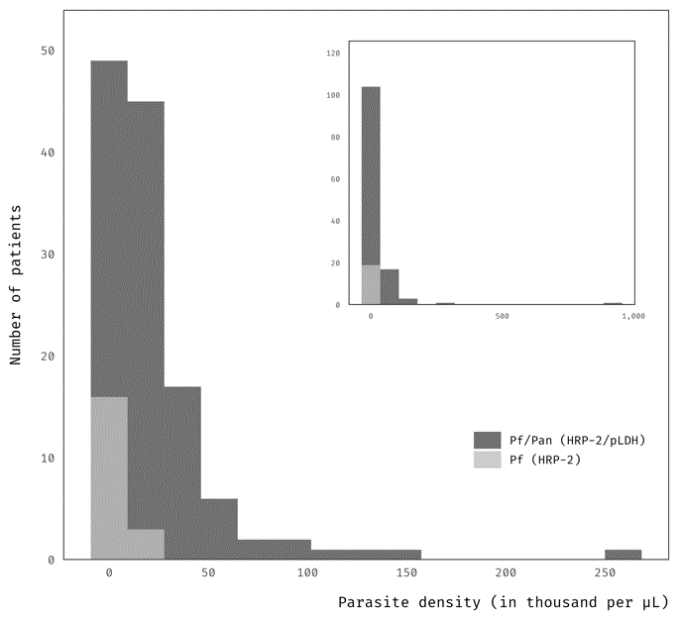

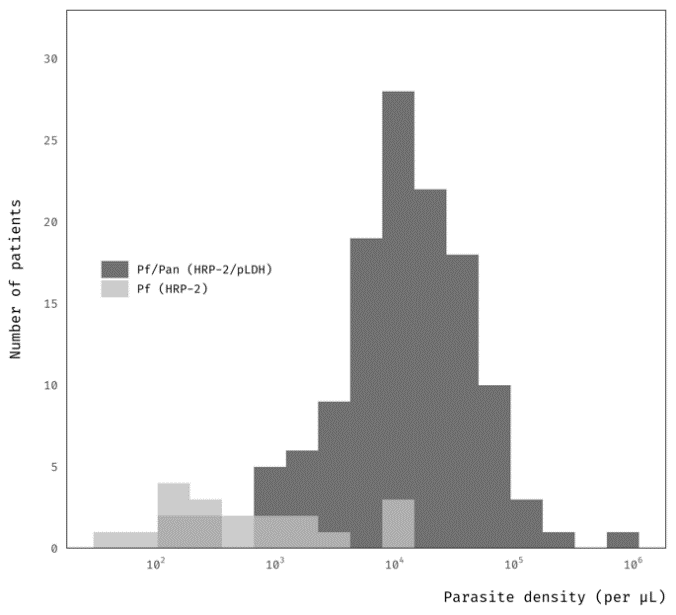


**(B)**

**(A)**

**Figure S1. Distributions of parasite density by test result.** (A) the original scale and (B) logarithmic scale. The inset plot in (A), a participant with a high parasite density of almost 1000000 per microlitre is shown.


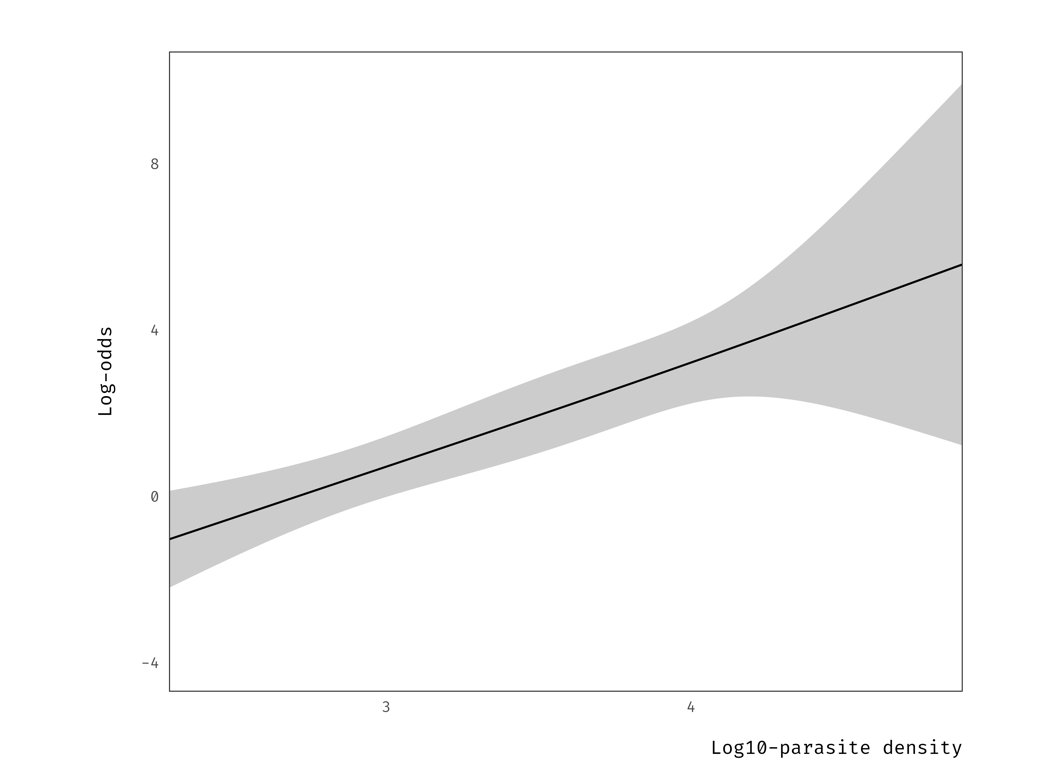


**Figure S2. Approximately linear relationship between log_10_-parasite density and the log-odds of Pf/Pan positivity.** The model was fitted using a logistic regression model that allows for non-linear relationship using restricted cubic splines with three knots. Grey area indicates the 95% confidence interval around the estimated log-odds. Horizontal axis is shown on the logarithmic scale.
